# NeuroPigPen: A Scalable Toolkit for Processing Electrophysiological Signal Data in Neuroscience Applications Using Apache Pig

**DOI:** 10.3389/fninf.2016.00018

**Published:** 2016-06-06

**Authors:** Satya S. Sahoo, Annan Wei, Joshua Valdez, Li Wang, Bilal Zonjy, Curtis Tatsuoka, Kenneth A. Loparo, Samden D. Lhatoo

**Affiliations:** ^1^Division of Medical Informatics, School of Medicine, Case Western Reserve UniversityCleveland, OH, USA; ^2^Electrical Engineering and Computer Science Department, School of Engineering, Case Western Reserve UniversityCleveland, OH, USA; ^3^Department of Neurology, School of Medicine, Case Western Reserve UniversityCleveland, OH, USA

**Keywords:** data flow language, Apache Pig, electrophysiological signal data, neuroscience, MapReduce

## Abstract

The recent advances in neurological imaging and sensing technologies have led to rapid increase in the volume, rate of data generation, and variety of neuroscience data. This “neuroscience Big data” represents a significant opportunity for the biomedical research community to design experiments using data with greater timescale, large number of attributes, and statistically significant data size. The results from these new data-driven research techniques can advance our understanding of complex neurological disorders, help model long-term effects of brain injuries, and provide new insights into dynamics of brain networks. However, many existing neuroinformatics data processing and analysis tools were not built to manage large volume of data, which makes it difficult for researchers to effectively leverage this available data to advance their research. We introduce a new toolkit called NeuroPigPen that was developed using Apache Hadoop and Pig data flow language to address the challenges posed by large-scale electrophysiological signal data. NeuroPigPen is a modular toolkit that can process large volumes of electrophysiological signal data, such as Electroencephalogram (EEG), Electrocardiogram (ECG), and blood oxygen levels (SpO_2_), using a new distributed storage model called Cloudwave Signal Format (CSF) that supports easy partitioning and storage of signal data on commodity hardware. NeuroPigPen was developed with three design principles: (a) Scalability—the ability to efficiently process increasing volumes of data; (b) Adaptability—the toolkit can be deployed across different computing configurations; and (c) Ease of programming—the toolkit can be easily used to compose multi-step data processing pipelines using high-level programming constructs. The NeuroPigPen toolkit was evaluated using 750 GB of electrophysiological signal data over a variety of Hadoop cluster configurations ranging from 3 to 30 Data nodes. The evaluation results demonstrate that the toolkit is highly scalable and adaptable, which makes it suitable for use in neuroscience applications as a scalable data processing toolkit. As part of the ongoing extension of NeuroPigPen, we are developing new modules to support statistical functions to analyze signal data for brain connectivity research. In addition, the toolkit is being extended to allow integration with scientific workflow systems. NeuroPigPen is released under BSD license at: https://sites.google.com/a/case.edu/neuropigpen/.

## Introduction

Rapid technological and methodological advances in sensing as well as recording neurological data in patients with epileptic seizures, stroke, and psychiatric disorders have dramatically improved the availability of high-resolution multi-modal neurological data for both biomedical research as well as patient care (Bargmann et al., 2014). These multi-modal datasets are playing a key role in neuroscience research efforts, for example they are advancing research in the brain connectivity networks using multiple data modalities representing both structural and functional networks (Swann et al., 2012; Wendling et al., 2016). The use of high-resolution magnetic resonance imaging (MRI) data together with sophisticated fiber tractography techniques has enabled the mapping of brain structural networks at multiple levels of granularity and these datasets can also be used to derive new information about fiber tract density and fiber tract orientation (Hagmann et al., 2006; Mukherjee et al., 2008). Similarly, high-resolution electrophysiological signals such as electroencephalogram (EEG) are providing new insights into brain functional connectivity networks (Isnard et al., 2004; Wendling et al., 2010). For example, intracranial depth electrodes implanted at precise brain locations using stereotactic placement approaches to record EEG (called SEEG) are being increasingly used in routine clinical care for evaluation and diagnosis of patients suffering from complex neurological disorders such as epilepsy (Schuele et al., 2012). Signal data from SEEG is used as gold standard during pre-surgical evaluation of epilepsy patients to identify brain tissues responsible for epileptic seizures, which can be removed during surgery and also to identify important brain regions such as the speech center that need to be protected during surgery (Lüders et al., 2012; Schuele et al., 2012).

The routine use of signal data in both patient care and biomedical research has led to rapid accumulation of large volumes of these datasets. For example, the epilepsy monitoring unit (EMU) at the University Hospitals Case Medical Center (UH-CMC) collects continuous multi-modal signal data from admitted patients over 5 days and it has collected more than 32 Terabytes (TB) of signal data in the past 4 years. The rate of data collection is increasing each year. The UH-CMC EMU is also part of the Center for Sudden and Unexpected Death in Epilepsy (SUDEP) Research (CSR), which is funded by the National Institute for Neurological Disorders and Stroke (NINDS), with 14 participating epilepsy centers across the USA and the UK (Lhatoo, 2014). The CSR project aims to collect and analyze signal, imaging, and related modalities of data from thousands of epilepsy patients. Similarly, the International Epilepsy Electrophysiology (IEEG) Portal hosts data from 1200 subjects as part of a multi-institutional initiative to create a repository of human and animal signal data, which can be used by researchers to advance epilepsy research as well as develop signal processing techniques (Kini et al., 2016). These two projects and similar initiatives in the neuroscience community (Marcus et al., 2011) highlight the need to develop scalable data management tools to effectively use large volumes of data to advance neuroscience research (Bargmann et al., 2014).

Storing, processing, and analyzing extremely large volumes of complex electrophysiological signal data requires development of sophisticated data partitioning, parallel computing, and distributed storage techniques that can effectively leverage several computing nodes for fast and scalable data processing applications. Current signal data processing tools were not developed using parallel processing techniques and they do not scale with increasing size of data. In addition, current file formats used to store signal data, such as the European Data Format (EDF; Kemp and Olivan, 2003), are not suitable for storage and processing of signal data in high performance distributed file systems. Therefore, new signal data format is needed to support partitioning data across several computing nodes. The development of these new tools also require significantly greater programming time as compared to traditional sequential data processing software tools to address new challenges inherent in distributed and parallel computing environments. For example, fault tolerance (both hardware or software failures) is a critical requirement for widespread deployment and use of neuroinformatics tools.

The open source Hadoop technology stack is being increasingly used to address scalability challenges faced by the scientific community for data and compute intensive tasks (Apache Hadoop, 2005). The MapReduce programming approach divides computational tasks into two recurring steps of Map and Reduce, which can be parallelized over hundreds or thousands of computing nodes (Dean and Ghemawat, 2010). Similar to Google MapReduce, the Hadoop implementation, which was developed by Yahoo!, can be deployed over inexpensive commodity hardware that allows easy expansion of the Hadoop cluster to scale with increasing volume of data (Borthakur et al., 2011). As part of the Apache Foundation project, there are number of Hadoop-based platforms that are being used to develop scalable software infrastructure for scientific computing. For example, the Hadoop Distributed File System (HDFS) allows reliable storage of large volume of data and supports data retrieval with high throughput for faster access (Shvachko et al., 2010). The Hive data warehouse platform is built on Hadoop to support analytical queries expressed in the HiveQL declarative language (Thusoo et al., 2010). In addition, HBase is a key-value store that uses HDFS to support database operations with high consistency and throughput for use in many social media and Web applications, such as Facebook (Borthakur et al., 2011). However, the use of Hadoop MapReduce for development of scalable data management tools is restricted to developers with advanced technical skills due to the complexity of parallel operations and multi-step data flows.

To address this challenge, the Apache Pig dataflow system was developed to allow users to easily compose multiple data processing functions into multi-steps dataflow applications, which are automatically compiled into MapReduce tasks by the Pig compiler (Gates et al., 2009). Pig also supports data manipulation functions by using SQL-like query constructs. The Pig system uses the Pig Latin programs to describe the data processing steps. Applications can use default Pig functions for data manipulation or create customized tasks called User Defined Functions (UDF) to support domain-specific data processing requirements, such as new signal data formats or data partitioning techniques. In this article, we describe the NeuroPigPen toolkit that consists of several UDFs, which were developed to process neurological signal data with built-in data partitioning, data transformation, and data processing functionalities. The NeuroPigPen UDFs are converted into MapReduce jobs by the Pig compiler and executed in a Hadoop cluster. In this article we describe the architecture of NeuroPigPen, the functionalities of the toolkit, and evaluate its performance using de-identified electrophysiological signal data collected at the UH-CMC EMU. The NeuroPigPen is available for download and use at: https://sites.google.com/a/case.edu/neuropigpen/ with user-friendly documentation, user manual, and licensing information.

## Materials and Methods

In this section, we describe the data processing requirements for neuroscience applications and functionalities supported by the NeuroPigPen toolkit modules. The NeuroPigPen toolkit is implemented using a modular approach, which allows them to be used both as part of an end-to-end workflow and as standalone tools.

### Role of Electrophysiological Signal Data in Neuroscience

Electrophysiological signal data such as EEG data, which is recorded using surface or intracranial electrodes, play a significant role in the evaluation of brain injuries, diagnosis of neurological disorders, and brain connectivity research (Sanei and Chambers, 2007). In contrast to other data modalities, SEEG data record brain functional activities at a fine level of granularity in both temporal and spatial scales, which is critical for clinical diagnosis and evaluation of patients with serious neurological disorders such as epilepsy. Epilepsy is the most common serious neurological disorder affecting about 50 million persons worldwide Epilepsy Foundation (2015) and signal data is used to identify the brain regions involved in epileptic seizures and evaluate the effect of anti-epileptic drugs. Seizure signals in epilepsy patients originate in one or more locations and involve additional brain regions, which together constitute a seizure network. The accurate characterization of seizure networks is an area of active research in neuroscience that often requires analyzing large volumes of signal data (Wendling et al., 2016). Signal data is analyzed and classified by domain experts to detect seizures and identify the signal features before, during, and after occurrence of seizure, which can be used for development of different types of devices to help epilepsy patients.

For example, there has been extensive work in developing signal processing techniques to automatically detect seizures, including use of time frequency approaches together with discrete wavelet transform and machine learning techniques over large signal datasets (Bayliss and Ballard, 2000). Electrophysiological signals as part of polysomnogram (PSG) data is also used in other biomedical domains such as sleep research (Redline et al., 2013). Therefore, there is a clear need for development of efficient and scalable tools for signal data that can be used in several biomedical applications. Similar to other time series data, electrophysiological signal data consists of discrete signal values and the associated timestamp values. EDF is the most widely used signal data representation and storage file format. An EDF file consists of two categories of metadata: (a) study-specific metadata, for example patient information, number of data records, start date and time of recording, and duration of data record; and (b) channel specific metadata, for example transducer type, number of samples per data record, and channel label.

**Figure 1:**
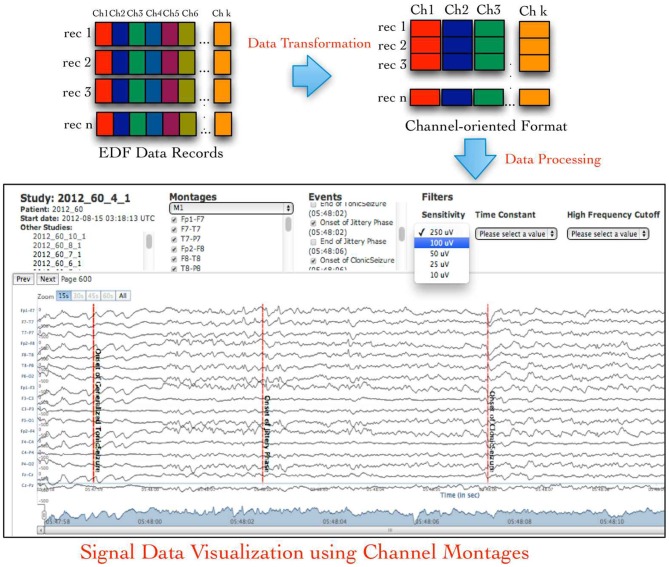
**European data format (EDF) files store signal data as contiguous samples recorded from all channels, which makes it difficult to easily access channel-specific signal data used in signal visualization and analysis applications.** The NeuroPigPen toolkit re-organizes the “sample-oriented” data in EDF files into contiguous “channel-oriented” data stored in cloudwave signal format (CSF) files to support neuroscience applications, such as Web-based signal visualization application.

An EDF file stores the signal data recorded from all recording channels during specific time duration (called an epoch) as binary values in sequential order, which is called an *EDF data record*. This sequential ordering of data from all channels in an EDF data record is not suitable for signal data visualization either for single channel or for combination of channels called a *signal montage*. Therefore, neuroinformatics applications need to transform the default EDF files into channel-oriented data records followed by additional data pre-processing for use in signal visualization or analysis tools (Figure 1 illustrates the default layout of an EDF file and desired channel-oriented format that is required by signal visualization applications; Jayapandian et al., 2013). The size of an EDF file is dependent on the number of recording channels, which may vary from few Megabytes (MB) to Gigabytes (GB). This unpredictability in size of EDF files makes it difficult for neuroinformatics applications, such as Web-based signal query and visualization applications (Jayapandian et al., 2013) to efficiently load and process EDF files. In addition, storage and analysis of large volumes of EDF files has become a critical challenge for the neuroscience research community due to the inherent limitations of traditional file systems.

Traditional file systems have several constraints, such as limitation on total number of addressable locations that make it difficult to store extremely large volume of data (e.g., storing more than 1 TB of data), difficulty in increasing the size of available storage as the size of data increases, and challenges associated with efficiently retrieving large datasets. In addition, user queries often require retrieval of specific segments of signal data corresponding to clinical events (e.g., epileptic seizures), which is extremely difficult in traditional file systems due to limitations of indexing approaches. These limitations are effectively addressed in HDFS (Shvachko et al., 2010). However, storing electrophysiological signal data in HDFS and making it available for use in various types of neuroscience applications requires additional data processing steps, including:

1. *Partitioning of signal data into smaller segments*: to allow easier storage in distributed file systems.
2. *Processing raw signal data and transforming them into suitable format*: including conversion of digital values to physical values and binary values to integer values; and
3. *Supporting clinical annotation of signal data using standardized terminology or vocabulary*: such as a domain ontology, which will reduce terminological heterogeneity and facilitate data integration as well as data retrieval operations.

We have developed a Javascript Object Notation (JSON)-based signal representation format called Cloudwave Signal Format (CSF) to support efficient partitioning and storage of signal data in HDFS (Jayapandian et al., 2015). CSF files enable storage of smaller sized signal data segments in self-descriptive files using both study-specific and channel-specific metadata together with ontology-based clinical annotation. CSF uses the JSON “attribute-value” pair structure with arbitrary levels of nesting (Crockford, 1999) to annotate segments of signal data with the metadata information as well as clinical events identified by clinicians, which are modeled in an epilepsy domain ontology called Epilepsy and Seizure Ontology (EpSO; Sahoo et al., 2014). In addition to epilepsy, the clinical annotations used in other neurological disease domains (e.g., Parkinson’s disease and Alzheimer’s disease) that are modeled in other disease-specific ontologies can also be used to annotate data in CSF files. A CSF file may contain arbitrary number of signal segments, each corresponding to user-defined time duration, for example 30 s epoch is the default duration for a signal segment in a CSF file. A CSF file currently stores two signal segments (each of 30 s duration) by default. We note that these parameters can be modified according to user or application requirements.

Each CSF file can be independently stored, accessed, and queried by software applications; therefore they can be easily stored in distributed file systems such as HDFS. The CSF files are also well suited for integration and used in a variety of neuroscience applications, including visualization tools and signal analysis software. These software applications can directly use the processed signal data as numeric values, which are organized in a channel-oriented layout. In our previous work, we developed a MapReduce program to process EDF files and generate CSF files (Jayapandian et al., 2015), however there were several limitations associated with our approach that constrained its use and deployment. The MapReduce program was difficult to deploy and required careful setup and configuration of various parameters. In addition, the users were required to have expertise in parallel programming to integrate the program in external software applications and there was limited support for composing multi-step data flows. Therefore, we developed the NeuroPigPen toolkit using the Pig data flow language to address these limitations. The NeuroPigPen modules support the generation of CSF files after partitioning, annotating, and transforming the signal data stored in an EDF file. In the next section, we describe the functionalities of the NeuroPigPen modules.

### NeuroPigPen Modules

**Figure 2:**
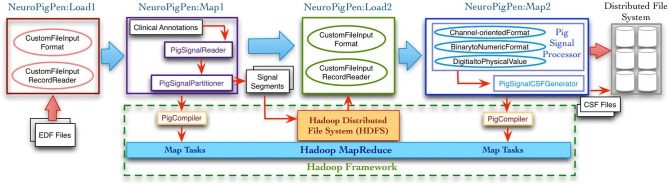
**The data processing workflow supported by the NeuroPigPen toolkit modules consists of multiple steps with EDF files as input and CSF files as output.** The Load functions in the toolkit extend the Hadoop FileInputFormat and FileInputRecordReader classes to support signal data. The Map functions in the toolkit are automatically compiled into MapReduce tasks by the Apache Pig compiler. The intermediate and final results are stored in Hadoop Distributed File System (HDFS), which provides a reliable and scalable storage platform for signal data.

Figure 2 shows the complete signal data processing workflow and the intermediate data processing steps supported by the NeuroPigPen modules, which are implemented as Pig UDFs. The Pig UDFs allow users to write customized load or data processing functions that can be used in Pig scripts to manage different types of data (Gates et al., 2009). The NeuroPigPen modules extend the Pig UDFs Application Programming Interface (API) to support specific functionalities required for processing signal data. The modules use HDFS to store the EDF files, the intermediate results, and the final CSF files, which ensure reliable storage of the signal data through HDFS replication feature. We describe the functionalities of the individual NeuroPigPen modules in the following sections:

#### PigSignalLoad

This module loads a set of EDF files from HDFS by instantiating the location of the EDF files on the Hadoop Data Nodes and extends two Hadoop APIs *InputFormat* and *RecordReader* to implement the *CustomFileFormat* and *CustomFileInputRecordReader* methods for reading and parsing the EDF files. These methods support partitioning the signal data into appropriately sized signal segments, which can be processed by the PigSignalReader module. The load module locates and identifies the EDF files and the associated annotation file (annotation files are stored separately according to the EDF specifications) using the “.edf” and “.txt” extensions for the file name, which is used by the PigSignalReader module to process the EDF files.

#### PigSignalReader

The PigSignalReader module parses, extracts, and aggregates the metadata information and signal data from the EDF files. In addition, it extracts the clinical annotations (e.g., occurrence of seizure events and spikes) together with the associated timestamp values and aggregates them with metadata values extracted from the EDF files. The metadata and signal data are stored in intermediate data structures using a “key-value” format, which allows the PigSignalPartitioner module to easily compute the offset values for data recorded by specific channels within and across EDF data records. In addition, the PigSignalProcessor module uses the metadata values stored in these “key-value” data structures to convert the digital signal values into the corresponding physical values. We note that the PigSignalReader module can be used as a standalone software tool by other neuroscience applications to parse and extract information from EDF files.

#### PigSignalPartitioner

The monolithic structure used to store signal data in an EDF file makes it difficult to store and process EDF files in HDFS, therefore the signal data needs to be partitioned into smaller segments that can be easily transferred across Hadoop Data nodes. HDFS stores data in fixed size blocks (64 MB by default in Apache Hadoop and 128 MB in the Cloudera distribution of Hadoop), which are distributed across multiple nodes in a cluster. However, there are no existing approaches that can be used to partition signal data in an EDF file. The PigSignalPartitioner module partitions the signal data in an EDF file into logical segments of fixed time duration, for example 30 s time duration (called *epochs*). The PigSignalPartitioner module allows users to specify the time duration for each partition as a configurable value based on application requirements. This feature allows neuroscience applications to flexibly create segments of signal data corresponding to variable time durations. After partitioning the signal data into smaller segments, the PigSignalPartitioner module adds the metadata and clinical annotations (extracted by PigSignalReader) to these segments for additional processing by the PigSignalProcessor module and generation of CSF file by the PigSignalCSFGenerator module.

#### PigSignalProcessor

The default layout for signal data in an EDF file involves contiguous storage of data recorded from all channels (e.g., EEG and ECG) within a specific time period as a single EDF data record. This data organization is not suitable for analyzing or visualizing data from a single channel or combination of channels. Therefore, the PigSignalProcessor module transforms the layout of signal data in a given signal segment into a channel-oriented layout. Data from each channel consists of different number of samples, for example signals that change frequently are recorded at a higher rate of sampling such as ECG. The PigSignalProcessor module uses the channel-specific metadata (extracted by the PigSignalReader) to compute the number of samples corresponding to each recording channel and then aggregates all the channel-specific data into a single data record. This channel-oriented layout stores signal data from each channel contiguously to support faster retrieval of data corresponding to single channel or combine data from different channels to compose a signal montage (Jayapandian et al., 2013).

The PigSignalProcessor module also converts the original signal data values, which are stored as binary values, into numeric values for use by signal visualization tools. This pre-processing step reduces the computational load of visualization application and supports improved response time for rendering large volumes of signal data. In addition, the PigSignalProcessor module converts the “digital” signal values (as recorded in the original EDF file) to “physical” values, which can be used for analysis of applications such as functional connectivity algorithms (Wendling et al., 2016). The conversion process uses a standard approach based on the digital and physical minimum as well as maximum values and stores them in suitable data structures for conversion into CSF file by the PigSignalCSFGenerator module.

#### PigSignalCSFGenerator

The PigSignalCSFGenerator module generates CSF files using signal data segments and aggregated metadata generated by the PigSignalPartitioner and PigSignalProcessor modules. In addition to the metadata values extracted from the EDF files, the PigSignalCSFGenerator computes additional metadata information to facilitate easier search, retrieval, and indexing of the CSF files, for example start and end time of individual signal segments and the sequence number of a signal segment based on its recording time. This allows data retrieval applications to efficiently locate signal segments corresponding to specific recording time in CSF files. It is important to note that the CSF format will continue to evolve to incorporate new metadata fields and the design of the PigSignalCSFGenerator makes it easy to modify and maintain it. The “self-descriptive” property of JSON “attribute-value” pairs allows neuroinformatics applications to maintain compatibility across different versions of CSF files.

**Figure 3:**
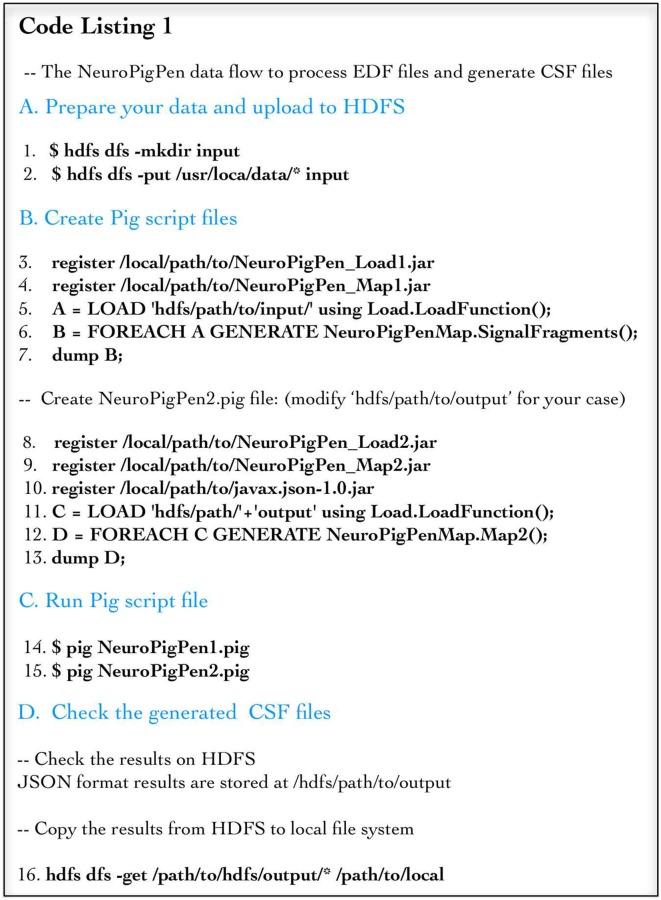
**Code Listing 1 shows the steps to be followed to use the NeuroPigPen modules using command line scripts for Pig.** The steps (shown using four sections) show loading the data into HDFS, creation of Pig script files, executing the Pig script files, and validating the output CSF files. Apache Pig scripts can be embedded and invoked from other programming language, such as Java and Python.

Code Listing 1 (Figure 3) shows the components of the NeuroPigPen toolkit together with the command line scripts used to register the NeuroPigPen UDFs, generate and execute the Pig scripts. For brevity, the comments and header information are not listed. The lines 1 and 2 in Section A transfer EDF files from a local storage location (e.g., personal computer or local server) to HDFS. The lines 3–7 in Section B register the first two Java Archive (jar) files corresponding to the load and Map functions. The lines 8–13 in Section B register the second set of jar files corresponding to the second load and Map functions. The lines 14 and 15 in Section C execute the two registered functions to process the EDF files stored in HDFS, where the UDFs are compiled into Map tasks by the Pig compiler. Line 16 in Section D validates the generation of CSF files. In the following section, we evaluate the NeuroPigPen toolkit with respect to its three design principles.

## Results

The NeuroPigPen was designed to incorporate three primary features of: (1) scalability to process and analyze large volumes of electrophysiological signal data; (2) ease of programming through use of high level programming constructs, which reduces the need to address parallelization, data partitioning, and inter-node communication challenges by developers; and (3) adaptability to different cluster configurations in terms of available computing nodes, which will allow research groups with small as well as large computing clusters to use the NeuroPigPen toolkit without the need to modify code. The NeuroPigPen toolkit meets these three design objectives. By leveraging the features of the MapReduce parallel programming approach and the high-level data flow programming design of Apache Pig, NeuroPigPen can be easily integrated in neuroinformatics software without compromising on computing performance. Similar to Apache Hadoop and Pig, the NeuroPigPen toolkit has been developed using Java, which makes it easily portable across heterogeneous computing platforms.

We systematically evaluated the scalability and adaptability of the NeuroPigPen toolkit using de-identified electrophysiological signal data collected at the UH-CMC EMU, which were stored as EDF files. We removed all Protected Health Information (PHI) data elements from the EDF files before using it to evaluate NeuroPigPen. The EDF files were processed over a 31-node Hadoop cluster at our High Performance Computing Cluster (HPCC) using Cloudera CDH 5.4 distribution. Each Data node in the Hadoop cluster has a dual Xeon E5450 3.0 GHz processor with eight cores per processor, 16 GB memory, and 2 TB disk storage. The Hadoop Name node has a dual Xeon 2.5 GHz E5-2450 processor with 16 cores, 64 GB memory, and 1 TB disk storage. The nodes are connected by 10 gigabits network connection. The HPCC is protected by institutional firewall with fine-grained access control to manage access to the de-identified signal data. We used a HDFS replication factor of 3 to store the datasets in the HPCC cluster. The results reported in this section are the average value of three executions with cold cache on the HPCC cluster.

### Evaluation of the Scalability and Adaptability of NeuroPigPen

**Figure 4:**
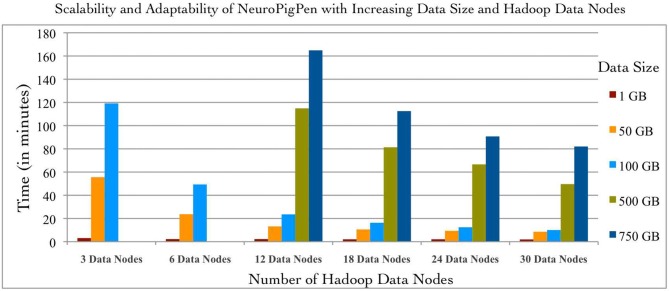
**The scalability and adaptability of the NeuroPigPen is demonstrated by processing five datasets with 1, 50, 100, 500, and 750 GB size over six different configuration of Hadoop Data nodes featuring 3, 6, 12, 18, 24, and 30 nodes.** The experiment results show that NeuroPigPen scales with increasing volumes of data and can be deployed over different sizes of Hadoop clusters.

The scalability of NeuroPigPen was tested using five datasets with sizes: 1, 50, 100, 500, and 750 GB. The two datasets of size 50 and 500 GB were selected to demonstrate the effect on performance of NeuroPigPen when the size of data increases by an order of magnitude. An EDF file is the “data input unit” for NeuroPigPen, that is, each EDF file is processed individually. The signal datasets consisted of EDF files with different sizes ranging from 248 MB to 19.6 GB to reflect the variability in size of individual EDF files collected in real world settings. The EDF files were stored in a single folder and the path to the folder was used as input for the PigSignalLoad module. Figure 4 shows the performance of NeuroPigPen with increasing size of data, which demonstrates the scalability of the toolkit as the size of data increases from 1 GB to a maximum of 750 GB. It is important to note that as the size of data is increased by an order of magnitude (50 to 500 GB), the time required for processing the data increases by less than an order of magnitude (from 8.57 to 49.63 min on 30 Hadoop Data nodes), which demonstrates the efficient performance of NeuroPigPen. In addition to scalability, the NeuroPigPen also meets it design objective for adaptability as Figure 4 shows that it effectively leverages available Hadoop Data nodes to reduce the time required to process the signal data.

This adaptability allows NeuroPigPen to be used in a variety of environments ranging from small clusters (often used by neuroscience research groups with limited financial resources) to large clusters. To systematically evaluate the adaptability of NeuroPigPen, we used six different configurations of Hadoop consisting of: 3, 6, 12, 18, 24, and 30 Data nodes. The six different configurations were evaluated by increasing the number of Data nodes available to NeuroPigPen in the HPCC cluster for fixed size of signal data. The results in Figure 4 show that the performance of NeuroPigPen improves with increasing number of Data nodes, we specifically note the improvement in performance as there is an order of magnitude increase in the number of Data nodes (3–30 nodes). The improvement in performance is more significant for larger datasets, for example there is an order of magnitude reduction in time performance for 100 GB dataset, as compared to smaller dataset such as 1 GB. We note that the 500 GB and 750 GB sized datasets could not be processed with less than 12 Data nodes. This highlights a physical constraint on the total size of signal data that can be processed on smaller-sized Hadoop clusters.

### Ease of Programming and Performance of Individual Map Functions

**Figure 5:**
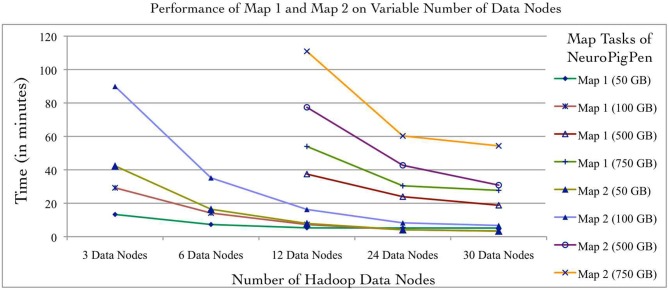
**The NeuroPigPen modules can be used as standalone applications, which will enable users to selectively use modules to support specific applications.** The Map1 function of NeuroPigPen consists of the PigSignalReader module and the Map2 function consists of the PigSignalPartitioner, PigSignalProcessor, and the PigSignalCSFGenerator modules. The comparative evaluation of the two Map functions uses the five datasets and six Hadoop configurations used in the first experiment. The performance evaluation shows that Map2 takes approximately twice the time to process a given dataset as compared to Map1 and this is consistent across different datasets as well as Hadoop configurations.

Individual NeuroPigPen modules can be embedded into other programming languages such as Java or Python to support complex control flows for processing and analyzing signal data. This allows NeuroPigPen to be used for composing data flows with complex constructs, including recursions, which are not directly supported by Pig. The modular feature of NeuroPigPen makes it easier for users and developers to integrate the whole toolkit or individual modules to process signal data. To evaluate the performance of the two Map functions in NeuroPigPen (as listed in the Code Listing 1, Figure 3), we recorded the time taken to process the data for individual Map functions using the six Hadoop Data node configurations and five datasets used in the previous experiment. Figure 5 shows that the Map2 function (in Code Listing 1, Figure 3) consisting of the PigSignalPartitioner, PigSignalProcessor, and PigSignalCSFGenerator modules require significantly more time to complete its execution as compared to Map1, which consists of the PigSignalReader module. The Map2 function takes three times longer to process 100 GB of data on three Data nodes (29.2 min) as compared to Map 1 (89.9 min) and about two times longer on 30 Data nodes (3.3 min as compared to 6.7 min). These results demonstrate that applications can use the PigSignalReader to parse EDF files without incurring the extra computational time required for generating CSF files by the Map2 function.

As part of our ongoing research in computing functional connectivity measures from SEEG data in epilepsy patients, we have integrated the NeuroPigPen modules into a computational workflow that uses EDF files as input and generates connectivity measures as output. The functional connectivity are computed using non-linear correlation coefficient to quantify the degree of co-occurrence of signal values *X(t)* and *Y(t)* recorded from two brain locations (mapped to the recording contacts on the intracranial electrodes) at *G*_X_ and *G*_Y_ where *t* is time of recording (Wendling et al., 2010). To support the extremely large volume of SEEG data in our project, the EDF files are processed using all the NeuroPigPen modules and the resulting smaller-sized CSF files are used as input to the module computing non-linear correlation coefficient measures. The integration of the NeuroPigPen modules with the functional connectivity workflow required minimal programming effort in terms of allowing the correlation coefficient measure module to process CSF files. The ease of using NeuroPigPen in the functional connectivity workflow demonstrates its suitability for use in other computational neuroscience applications. For example, analysis involving correlations and granger causality over small segments of EDF files storing sleep recording can be supported by NeuroPigPen modules with minimal modification.

### Related Work

The evaluation results for NeuroPigPen demonstrate that it is a practical toolkit for use in neuroscience applications. There are several initiatives to use Hadoop technologies to process and analyze neuroscience data, including use of Hadoop Spark for mapping brain activity (Freeman et al., 2014), and signal data processing using Graphic Processing Unit (GPU; Chen et al., 2011). There has been work on use of MapReduce to process large volumes of EEG data (Wang et al., 2012) and for signal analysis (Wu and Huang, 2009; we refer to Nguyen et al., 2011 for an overview of using Hadoop technologies for clinical signal data). We are not aware of an existing Apache Pig-based neuroscience data processing library that can be compared to the functionalities and features of the NeuroPigPen toolkit. The scalability, adaptability, and the ability to use individual NeuroPigPen modules to support different types of signal processing makes this toolkit an important resource for the growing neuroinformatics community that uses Hadoop technologies. As part of our ongoing work, we are extending the functionalities supported by the NeuroPigPen modules to include several statistical measures, such as computing correlation functions between signal data. These statistical measures are often used to compute functional connectivity measures from EEG signal data as part of brain connectivity research (Wendling et al., 2010). In the next section, we discuss the broader impact and current limitations of the NeuroPigPen toolkit.

## Discussion

The primary advantage of the NeuroPigPen toolkit as compared to hand crafted MapReduce applications is the use of high-level data flow programming constructs defined in Apache Pig without compromising the performance of the toolkit in terms of scalability. However, the generic MapReduce tasks generated by the Apache Pig compiler are not optimized for processing electrophysiological signal data, therefore the NeuroPigPen may not be as efficient as hand crafted MapReduce programs. We propose to perform a systematic comparison of NeuroPigPen with MapReduce programs in the future to systematically characterize the difference in performance. This comparison will help users in making a decision regarding the use of NeuroPigPen for easy integration and availability or development of hand crafted MapReduce programs. In addition, there are well-known issues of latency associated with initialization of Hadoop MapReduce applications (Pavlo et al., 2009), which also affect the performance of NeuroPigPen modules. This latency is especially noticeable for smaller datasets as compared to large datasets as the data processing time for large datasets tends to dominate in comparison to the initialization time for Hadoop applications. There are several approaches that can be used to address the issue of latency, including use of indexes and data pre-fetching techniques. Applications that do not perform well on MapReduce architecture will also not perform well with NeuroPigPen. Therefore, neuroscience applications need to consider this aspect before making a decision regarding the use of NeuroPigPen toolkit.

Integration of NeuroPigPen modules with scientific workflow systems, such as Taverna (Hull et al., 2006) and Kepler (Ludäscher et al., 2006), will allow wider adoption of NeuroPigPen by the neuroscience research community for processing large datasets. Scientific workflows are widely used in the bioinformatics and medical informatics community to automate data processing across distributed computing resources with support for failure recovery and ability to collect provenance metadata for scientific reproducibility (Missier et al., 2010). Although, there is a clear synergy between the NeuroPigPen toolkit and scientific workflow systems, we need to address the lack of support for workflow system in NeuroPigPen modules. For example, we need to develop remotely accessible APIs, such as Representational State Transfer (RESTful) Web services, to allow workflow engines to invoke the NeuroPigPen modules. In addition, there is a need to use CSF as the common data representation format by workflow engines to support exchange of signal data between the workflow systems and neuroscience applications. Therefore, as part of our future work we plan to develop RESTful APIs for NeuroPigPen modules. This will allow scientific workflow systems to leverage the advantages of Hadoop framework (e.g., scalability) to process and analyze neuroscience data especially in the broader context of combined multi-modal recordings. We are also developing new NeuroPigPen modules to support imaging data used in neuroscience application, for example, characterization of cognitive and neural correlates of mathematics problem solving using fMRI.

## Conclusion

In this article, we introduced the NeuroPigPen toolkit to address the need of the neuroinformatics community to process large volumes of electrophysiological signal data, which is used in several neuroscience applications. For example, characterization of seizure networks in epilepsy patients and computing functional connectivity network. We demonstrated the usefulness of this toolkit and its modularity, which enables NeuroPigPen to be used in existing software applications and leverage the advantages of Hadoop technologies. The toolkit was designed to allow developers and users to use a high-level data flow programming approach to construct signal data processing workflows in contrast to developing hand crafted MapReduce programs or using Message Passing Interface (MPI), which requires developers to address the complexities associated with parallel programming. In addition, we demonstrated that NeuroPigPen meets its three design objectives of scalability, adaptability, and ease of use by evaluating its performance with 750 GB signal data over variable number of Hadoop Data nodes. The toolkit was developed using the Java programming language, therefore it is portable across heterogeneous computing environments. As part of our ongoing work, we are adding new modules to the NeuroPigPen toolkit to support additional features such as computing statistical measures for brain connectivity applications and potential integration with scientific workflow systems.

## Author Contributions

AW, LW, JV, SSS developed NeuroPigPen algorithm, architecture, and modules. SSS designed the evaluation experiments that were implemented by AW, LW, and JV. SDL, BZ, CT, and SSS identified the functions to be supported by NeuroPigPen in context of epilepsy clinical research, signal visualization, and analysis functions. SDL and KAL validated the features of signal processing steps and its utility in signal analysis applications. All authors contributed to preparation of the article, figures, and charts.

## Funding

This work is supported in part by the National Institutes of Biomedical Imaging and Bioengineering (NIBIB) Big Data to Knowledge (BD2K) grant (1U01EB020955) and the National Institutes of Neurological Disorders and Stroke (NINDS) Center for SUDEP Research grant (1U01NS090407-01).

## Conflict of Interest Statement

The authors declare that the research was conducted in the absence of any commercial or financial relationships that could be construed as a potential conflict of interest.
